# Supplementary material for: On model-based time trend adjustments in platform trials with non-concurrent controls
Source: BMC Med Res Methodol. 2022 Aug 15;22:228. doi: 10.1186/s12874-022-01683-w (PMC9380382; doi:10.1186/s12874-022-01683-w)
Supplement: Supplementary file 1 — Additional file 1 Supplementary document with technical derivations, proofs, and additional simulation study results. [file 12874_2022_1683_MOESM1_ESM.pdf]

Supplementary material for "On model-based time trend adjustments in platform trials with non-concurrent controls"

Marta Bofill Roig, Pavla Krotka, Carl-Fredrik Burman, Ekkehard Glimm, Stefan M. Gold, Katharina Hees, Peter Jacko, Franz Koenig, Dominic Magirr, Peter Mesenbrink, Kert Viele, and Martin Posch*

Contents

A Weights derivation **2**

B Example weights for a particular case **4**

C Properties of the estimators and tests under model-based period-wise adjustments **4**

D Impact of time trends on the variance estimation in the linear model **5**

E Results of the simulation study **6**
- E.1 Continuous endpoints . . . 6
  - E.1.1 Equal time trends . . . 7
  - E.1.2 Different time trends . . . 10
- E.2 Binary endpoints . . . 12
  - E.2.1 Equal time trends . . . 13
  - E.2.2 Different time trends . . . 16

F Additional simulations **21**
- F.1 Comparison of randomization procedures and patient entry time schemes . . . 21
  - F.1.1 Data generation . . . 22
  - F.1.2 Randomization procedures . . . 22
  - F.1.3 Results . . . 22
- F.2 Platform trials with three periods . . . 23
  - F.2.1 Data generation . . . 23
  - F.2.2 Results . . . 24

*martin.posch@meduniwien.ac.at

In this supplementary material, we present the derivation of the weights presented in Section 3.1.1 of the main paper (in Section A), together with an example of weights computation (in Section B), and proofs of the properties of the estimators explained in Section 3.1.2 (in Section C). Moreover, we report further remarks on the impact of time trends on the variance estimation in Section D.
We also present here the results of the simulation study and results from additional simulations (in Sections E and F, respectively). Regarding the simulation results in Section E, we show the bias and root mean squared error of the treatment effect estimators in the presence of time trends. We also present the results for those models in Section 4 of the main paper.
Throughout this document, we use the following abbreviations for the models: ALLTC refers to models using all treatment data and control (see equations 1 and 6 in the main paper), ALLTCI to models using all treatment data and control with the interaction between time and treatment arm (see equations 2 and 7), and TC to models using only data from one treatment arm and the control (see equations 5 and 8); the suffixes Step/Linear indicate whether models adjust time linearly or step-wise.

A Weights derivation

We may obtain the estimates for the linear model by forming the design matrix X, with one row for each patient and with columns for the intercept, arm 1, arm 2, and 2nd time period. If the interaction is included, then X contains an additional column for interaction of arm 1 and the 2nd time period. The matrix elements are 0's and 1's, where 1 indicates that a particular patient matches up to a particular feature. For instance, for the model without interaction, we have

$$X = \begin{matrix} n_{0,1} \\ n_{1,1} \\ n_{0,2} \\ n_{1,2} \\ n_{2,2} \end{matrix} \begin{pmatrix} 1 & 0 & 0 & 0 \\ 1 & 1 & 0 & 0 \\ 1 & 0 & 0 & 1 \\ 1 & 1 & 0 & 1 \\ 1 & 0 & 1 & 1 \end{pmatrix}$$

where the row labels indicate their multiplicities.
The vector estimate of all parameters is $(X^\top X)^{-1}X^\top y$, where y is the vector of patient responses. Thus each parameter estimate is a linear combination of the observations. Our interest lies in the estimate of the arm 2 treatment effect

$$\hat{\theta}_2 = \sum_j v_j y_j$$

where v_j is the $[3, j]$ element of $(X^\top X)^{-1}X^\top$ (since row 3 corresponds to the treatment effect of arm 2) and y_j is the response for the jth patient. Note that all patients with the same covariate values (and thus identical rows of the X matrix) will have the same v_j values, allowing us to collect terms and rewrite

$$\hat{\theta}_2 = \sum_{k,s} w_{k,s}\bar{y}_{k,s}$$

where $w_{k,s} = n_{k,s}v_{k,s}$ and $v_{k,s}$ is the common value of v_j for all patients in arm k and time period s. Note that the weighted average is determined entirely by the sample sizes in each arm and time period, not the responses for those patients.
If there are no arm 2 patients in time period 1 ($n_{2,1} = 0$), and the interaction model is used, then the weighted average is extremely simple, with all $w_{k,s} = 0$ except for $w_{0,2} = -1$ and $w_{2,2} = 1$. (We omit the mathematical derivation.) In other words, the estimated treatment is simply the difference in means in the 2nd time period, resulting in the concurrent controls analysis.
If there are no arm 2 patients in time period 1 ($n_{2,1} = 0$), and the non-interaction model is used, then the weights can be obtained as follows. Let $\boldsymbol{v}$ be a column vector created by concatenating individual patient weights v_j so that the first $n_{0,1}$ elements correspond to patients on arm 0 in period 1, the following $n_{1,1}$ elements correspond to patients on arm 1 in period 1, the following $n_{0,2}$ elements correspond to patients on arm 0 in period 2, the following $n_{1,2}$ elements correspond to patients on arm 1 in period 2, the final $n_{2,2}$

elements correspond to patients on arm 2 in period 2. Since the 3rd row of $(X^\top X)^{-1}X^\top$ corresponds to row vector $\boldsymbol{v}^\top$, after taking a transpose we have that the 3rd column of $X(X^\top X)^{-1}$ corresponds to vector $\boldsymbol{v}$. Also, observe that

$$\begin{array}{c} \begin{matrix} n_{0,1} & n_{1,1} & n_{0,2} & n_{1,2} & n_{2,2} \end{matrix} \\ \begin{pmatrix} 1 & 0 & 0 & 0 & 0 \\ 0 & 1 & 0 & 0 & 0 \\ 0 & 0 & 1 & 0 & 0 \\ 0 & 0 & 0 & 1 & 0 \\ 0 & 0 & 0 & 0 & 1 \end{pmatrix} \end{array} \boldsymbol{v} = \begin{pmatrix} w_{0,1} \\ w_{1,1} \\ w_{0,2} \\ w_{1,2} \\ w_{2,2} \end{pmatrix}$$

where the column labels indicate column multiplicities. Then,

$$X(X^\top X)^{-1} \begin{pmatrix} 0 \\ 0 \\ 1 \\ 0 \end{pmatrix} = \boldsymbol{v}$$

is equivalent to

$$\begin{array}{c} \begin{matrix} n_{0,1} & n_{1,1} & n_{0,2} & n_{1,2} & n_{2,2} \end{matrix} \\ \begin{pmatrix} 1 & 0 & 0 & 0 & 0 \\ 0 & 1 & 0 & 0 & 0 \\ 0 & 0 & 1 & 0 & 0 \\ 0 & 0 & 0 & 1 & 0 \\ 0 & 0 & 0 & 0 & 1 \end{pmatrix} \end{array} X(X^\top X)^{-1} \begin{pmatrix} 0 \\ 0 \\ 1 \\ 0 \end{pmatrix} = \begin{pmatrix} w_{0,1} \\ w_{1,1} \\ w_{0,2} \\ w_{1,2} \\ w_{2,2} \end{pmatrix}$$

It is easy to see that

$$\begin{array}{c} \begin{matrix} n_{0,1} & n_{1,1} & n_{0,2} & n_{1,2} & n_{2,2} \end{matrix} \\ \begin{pmatrix} 1 & 0 & 0 & 0 & 0 \\ 0 & 1 & 0 & 0 & 0 \\ 0 & 0 & 1 & 0 & 0 \\ 0 & 0 & 0 & 1 & 0 \\ 0 & 0 & 0 & 0 & 1 \end{pmatrix} \end{array} X = \begin{pmatrix} n_{0,1} & 0 & 0 & 0 \\ n_{1,1} & n_{1,1} & 0 & 0 \\ n_{0,2} & 0 & 0 & n_{0,2} \\ n_{1,2} & n_{1,2} & 0 & n_{1,2} \\ n_{2,2} & 0 & n_{2,2} & n_{2,2} \end{pmatrix}$$

and that

$$X^\top X = \begin{pmatrix} N & n_{1,1}+n_{1,2} & n_{2,2} & n_{0,2}+n_{1,2}+n_{2,2} \\ n_{1,1}+n_{1,2} & n_{1,1}+n_{1,2} & 0 & n_{1,2} \\ n_{2,2} & 0 & n_{2,2} & n_{2,2} \\ n_{0,2}+n_{1,2}+n_{2,2} & n_{1,2} & n_{2,2} & n_{0,2}+n_{1,2}+n_{2,2} \end{pmatrix}$$

It can be shown that

$$(X^\top X)^{-1} \begin{pmatrix} 0 \\ 0 \\ 1 \\ 0 \end{pmatrix} = \begin{pmatrix} -\frac{1}{n_{0,1}}\varrho \\ \left(\frac{1}{n_{0,1}} + \frac{1}{n_{1,1}}\right)\varrho \\ \frac{1}{n_{2,2}} + \left(\frac{1}{n_{0,1}} + \frac{1}{n_{1,1}} + \frac{1}{n_{1,2}}\right)\varrho \\ -\left(\frac{1}{n_{1,1}} + \frac{1}{n_{1,2}}\right)\varrho \end{pmatrix}$$

where

$$\varrho = \frac{\frac{1}{n_{0,2}}}{\frac{1}{n_{0,1}} + \frac{1}{n_{1,1}} + \frac{1}{n_{0,2}} + \frac{1}{n_{1,2}}}$$

Therefore,

$$\begin{pmatrix} w_{0,1} \\ w_{1,1} \\ w_{0,2} \\ w_{1,2} \\ w_{2,2} \end{pmatrix} = \begin{pmatrix} -\varrho \\ \varrho \\ \varrho - 1 \\ -\varrho \\ 1 \end{pmatrix}$$

B Example weights for a particular case

If equal randomisation is employed also for arm 2, that is, $n_{0,2} = n_{1,2} = n_{2,2}$, then we can express the weights using the ratio of period sample sizes. Denoting by $\varphi = N_1/N_2$ and taking into account that $\omega = \frac{3}{2}\varphi$, we can rewrite the weights' matrix as

$k\backslash s$	1	2
0	$-\frac{1}{2} + \frac{1}{3\varphi+2}$	$-\frac{1}{2} - \frac{1}{3\varphi+2}$
1	$\frac{1}{2} - \frac{1}{3\varphi+2}$	$-\frac{1}{2} + \frac{1}{3\varphi+2}$
2	0	1

thus obtaining $\tilde{\theta}_2 = (\bar{y}_{2,2} - \bar{y}_{0,2}) + \left(\frac{1}{2} - \frac{1}{3\varphi+2}\right)[(\bar{y}_{1,1} - \bar{y}_{0,1}) - (\bar{y}_{1,2} - \bar{y}_{0,2})]$. For instance, if $N_1/N_2 = 2/3$, then we have

$k\backslash s$	1	2
0	−0.25	−0.75
1	0.25	−0.25
2	0	1

, and $\tilde{\theta}_2 = (\bar{y}_{2,2} - \bar{y}_{0,2}) + 0.25\,[(\bar{y}_{1,1} - \bar{y}_{0,1}) - (\bar{y}_{1,2} - \bar{y}_{0,2})]$;

C Properties of the estimators and tests under model-based period-wise adjustments

Model (4) in the paper, can be written as

$$E(g(Y_j)\,|t_j\,) = \theta_{k_j} + f(t_j) \tag{1}$$

where $k_j = 0, 1, 2$ is the treatment that patient j was randomised to. We set $\theta_0 := \eta_0$ for convenience of notation. Here, and in all subsequent derivations, we always condition on the observed k_j, but treat t_j as random.

We are considering 2 periods: $P = 1$ with $k_j = 0, 1$ and $P = 2$ with $k_j = 0, 1, 2$. Within period, t_j does not depend on k_j due to randomisation. For period 2 alone, we use the model

$$E(g(Y_j)\,|P = 2\,) = \mu_0 + \nu + \theta_{k_j}. \tag{2}$$

This arises from the conditional model in (1) via

$$E(y_j\,|P = 2\,) = E(E(y_j\,|t_j, P = 2\,)) = E(f(t_j)|P = 2) + \theta_{k_j} = \mu_0 + \nu + \theta_{k_j}.$$

Here, $E(f(t_j)|P = 2)$ is the same for all j due to randomisation and $E(f(t_j)|P = 2) =: \mu_0 + \nu$ is just a naming convention.

For period 1 alone, we use the model

$$E(g(Y_j)\,|P = 1\,) = \mu_0 + \theta_{k_j}. \tag{3}$$

This arises from the conditional model in (1) via

$$E(g(Y_j)\,|P = 1\,) = E(E(g(Y_j)\,|t_j, P = 1\,)) = E(f(t_j)|P = 1) + \theta_{k_j} = \mu_0 + \theta_{k_j}.$$

Here, $E(f(t_j)|P = 1)$ is the same for all j due to randomisation and $E(f(t_j)|P = 1) =: \mu_0$ is a naming convention. Taken together, we have $\mu_0 = E(f(t_j)|P = 1)$ and $\nu = E(f(t_j)|P = 2) - E(f(t_j)|P = 1)$.

From these considerations, it is obvious that (if we are not interested in the functional form of $f(t_j)$), we can use the "unconditional-within-period" model characterised by (3) and (2) for estimation of θ_{k_j} in the conditional model (1). The estimate is unbiased in the linear case where $g()$ is the identity function. In the logistic regression model, this estimate is asymptotically unbiased - just like the point estimates from a correctly specified model. Regarding precision, however, it may be advantageous to estimate $f(t_j)$ to refine the estimate of θ_{k_j}.

Regarding variance estimation, the asymptotic unbiasedness of the parameter estimates in the logistic regression model carries over to the variance estimates, since these are also functions of the estimated response probabilities. In the linear model, where the variance is modelled by an additional separate parameter, however, biases in variance estimation can arise if the residual variance changes in time and the randomisation ratio between control and test treatment changes in time. Additional remarks on these topics can be found in the next section.

D Impact of time trends on the variance estimation in the linear model

We assume that within period ($P = q$), the responses follow the model $y_j|T = t_j, P = q \sim N(f(t_j) + \theta_{k_j}, \sigma^2_{y\cdot T}(t_j))$. Hence, the variance is considered a function of time. We also assume that within period q, the time t_j at which patient j's response y_j is observed, is an independent, identically distributed (i.i.d.) sample from T_q. This is fulfilled if patients randomly enter the study at any point in time during the recruitment period and are randomized to a treatment. The distribution of T_q does not depend on the treatment k_j, but otherwise we leave it as unspecified. In particular, it might be different for $P = 1$ and $P = 2$. We have

$$E(y_j|P = q) = E(E(y_j|T_q, P = q)) = E(f(T_q)) + \theta_{k_j} =: \mu_q + \theta_{k_j}$$

and

$$\begin{aligned} var(y_j|P = q) &= var(E(y_j|T_q, P = q)) + E(var(y_j|T_q, P = q)) = \\ &var(f(T_q) + \theta_{k_j}) + E(\sigma^2_{y\cdot T}(T_q)) = var(f(T_q)) + E\left(\sigma^2_{y\cdot T}(T_q)\right). \end{aligned}$$

Thus, with $\sigma^2_q = var(f(T_q)) + E\left(\sigma^2_{y\cdot T}(T_q)\right)$, we have that

$$y_j|P = q \sim N(\mu_q + \theta_{k_j}, \sigma^2_q).$$

Within period q, the condition that $y_j|P = q$ are i.i.d. normally distributed is met. For $P = 1$ and $P = 2$, however, we have different variances. This can be addressed by fitting the model $y_j|P = q \sim N(\mu_q + \theta_{k_j}, \sigma^2_q)$ with $\mu_q = \mu_0 + \nu \cdot I(P = 2)$.
The (ML- or REML-)estimate $\hat{\theta}_k$ from this model will fulfill that $\sqrt{n}(\hat{\theta}_k - \theta)$ is asymptotically normal. Hence, we see that the step model which accounts for the time trend by introducing a period effect can also account for time-dependent changes in the variance by likewise modeling variance with a step function for period.
Regarding precision of the estimate, there will be a loss relative to knowledge of $f(\cdot)$: If we know $f(\cdot)$, then by conditioning on the observed t_j, we can eliminate $var(f(T_q))$ from σ^2_q.
A curious by-product of the above consideration is that we do not need to know $\sigma^2_{y\cdot T}(t_j)$ either. In practice, it might often be assumed that this does not depend on t_j. If we know that $\sigma^2_{y\cdot T}(t_j) = \sigma^2_{y\cdot T}$ and also know $f(\cdot)$, then $\sigma^2 = \sigma^2_q = \sigma^2_{y\cdot T}$ arises. If we do not know $f(\cdot)$, however, the assumption $\sigma^2_{y\cdot T}(t_j) = \sigma^2_{y\cdot T}$ does not prevent σ^2_q from being different for $q = 1$ and $q = 2$, so we might as well relax this assumption.

Let us now consider the case where it is (possibly erroneously) assumed that the residual variance is the same in the two periods. Hence, we have stochastically independent observations $y_j \sim N\left(\mathbf{x}_j'\boldsymbol{\theta}, \sigma^2_j\right)$, $j = 1, \ldots, n$. For the sake of estimation, it is assumed that $\sigma^2_j = \sigma^2$, but in reality, they might be different, e.g. depend on period of recruitment.
Let $\mathbf{X} = (\mathbf{x}_1, \ldots, \mathbf{x}_n)'$ be the $n \times p$-design matrix. We estimate $\boldsymbol{\theta}$ by $\hat{\boldsymbol{\theta}} = (\mathbf{X}'\mathbf{X})^{-1}\mathbf{X}'\mathbf{y}$ such that

$$\hat{\boldsymbol{\theta}} \sim N\left(\boldsymbol{\theta}, (\mathbf{X}'\mathbf{X})^{-1}\mathbf{X}'\boldsymbol{\Sigma}\mathbf{X}(\mathbf{X}'\mathbf{X})^{-1}\right)$$

where $\boldsymbol{\Sigma} = Diag(\sigma^2_j)_{j=1,\ldots,n}$. The residual error variance is estimated by

$$s^2 = \frac{1}{n}\mathbf{y}'\left(\mathbf{I}_n - \mathbf{X}(\mathbf{X}'\mathbf{X})^{-1}\mathbf{X}'\right)\mathbf{y} = \frac{1}{n}\sum_{j=1}^{n}\left(y_j - \mathbf{x}_j'\hat{\boldsymbol{\theta}}\right)^2.$$

If $n \to \infty$, the residuals $y_j - \mathbf{x}_j'\hat{\boldsymbol{\theta}}$ become asymptotically stochastically independent with variance σ_j^2 and hence s^2 converges to $\frac{1}{n}\sum_{j=1}^{n}\sigma_j^2 =: \bar{\sigma}^2$.
Assume now that we want to test a hypothesis $H_0 : \mathbf{h}'\boldsymbol{\theta} = 0$. We use

$$t = \frac{\mathbf{h}'\hat{\boldsymbol{\theta}}}{\sqrt{s^2 \cdot \mathbf{h}'\left(\mathbf{X}'\mathbf{X}\right)^{-1}\mathbf{h}}}.$$

Since the true variance of $\mathbf{h}'\hat{\boldsymbol{\theta}}$ is $\mathbf{h}'\left(\mathbf{X}'\mathbf{X}\right)^{-1}\mathbf{X}'\boldsymbol{\Sigma}\mathbf{X}\left(\mathbf{X}'\mathbf{X}\right)^{-1}\mathbf{h}$, $t \sim N(0,1)$ holds asymptotically under H_0 if

$$\bar{\sigma}^2 \cdot \mathbf{h}'\left(\mathbf{X}'\mathbf{X}\right)^{-1}\mathbf{h} = \mathbf{h}'\left(\mathbf{X}'\mathbf{X}\right)^{-1}\mathbf{X}'\boldsymbol{\Sigma}\mathbf{X}\left(\mathbf{X}'\mathbf{X}\right)^{-1}\mathbf{h}. \tag{4}$$

Hence, if we are able to find a parametrization of the model in which we show that

$$\mathbf{X}'\boldsymbol{\Sigma}\mathbf{X} = \bar{\sigma}^2 \cdot \mathbf{X}'\mathbf{X}, \tag{5}$$

then $t \sim N(0,1)$ holds asymptotically under H_0.
Since $\boldsymbol{\Sigma}$ is a diagonal matrix, we have $\mathbf{X}'\boldsymbol{\Sigma}\mathbf{X} = \sum_{j=1}^{n}\sigma_j^2 \cdot \mathbf{x}_j\mathbf{x}_j'$ and $\mathbf{X}'\mathbf{X} = \sum_{j=1}^{n}\mathbf{x}_j\mathbf{x}_j'$. A simple example where this holds is the following: Assume a two-group comparison $H_0 : \mu_1 - \mu_0 = 0$. Then

$$\mathbf{X} = \begin{pmatrix} \mathbf{1}_{n_0} & \mathbf{0}_{n_0} \\ \mathbf{0}_{n_1} & \mathbf{1}_{n_1} \end{pmatrix}.$$

Furthermore, $\mathbf{x}_j\mathbf{x}_j' = \begin{pmatrix} 1 & 0 \\ 0 & 0 \end{pmatrix}$ for a patient in group 0 and $\mathbf{x}_j\mathbf{x}_j' = \begin{pmatrix} 0 & 0 \\ 0 & 1 \end{pmatrix}$ for a patient in group 1. Thus, $\mathbf{X}'\mathbf{X} = \begin{pmatrix} n_0 & 0 \\ 0 & n_1 \end{pmatrix}$ and

$$\mathbf{X}'\boldsymbol{\Sigma}\mathbf{X} = \left(\sigma_1^2 + \ldots + \sigma_{n_0}^2\right)\begin{pmatrix} 1 & 0 \\ 0 & 0 \end{pmatrix} + \left(\sigma_{n_0+1}^2 + \ldots + \sigma_n^2\right)\begin{pmatrix} 0 & 0 \\ 0 & 1 \end{pmatrix}.$$

Hence, $\mathbf{X}'\boldsymbol{\Sigma}\mathbf{X} = \bar{\sigma}^2\mathbf{X}'\mathbf{X}$ if

$$(\sigma_1^2 + \ldots + \sigma_{n_0}^2)/n_0 = \left(\sigma_{n_0+1}^2 + \ldots + \sigma_n^2\right)/n_1 = \bar{\sigma}^2.$$

This example corresponds to the intuition that if we want to compare the means of two groups and these groups share the same average variance, then the t-test defined by t asymptotically keeps the type I error. From this consideration, it is also clear that examples where equation (5) does not hold can be constructed, e.g. if the majority of observations on treatment 0 is from period 1, the majority of observations from treatment 1 is from period 2 and the variance is larger in period 2 than in period 1.

E Results of the simulation study

All simulations were carried out using 100,000 replications per scenario. At each iteration, all models were fitted to the same simulated datasets. This explains common patterns regarding the smoothness of the estimates with respect to time trends (see, for instance, Figure S6).

E.1 Continuous endpoints

In what follows, we present additional results for continuous endpoints in the presence of time trends, which are either equal across all arms (Section E.1.1), or equal in the control group and treatment 2 but different in treatment 1 (Section E.1.2). We consider the time trend's patterns: linear, step-wise and inverted-U. For the inverted-U trend, we consider three different settings depending on the point at which the trend changes from positive to negative. This point can be either in the middle of period 1 ($N_p = 125$), between the two periods ($N_p = 250$) or in the middle of period 2 ($N_p = 500$).

E.1.1 Equal time trends

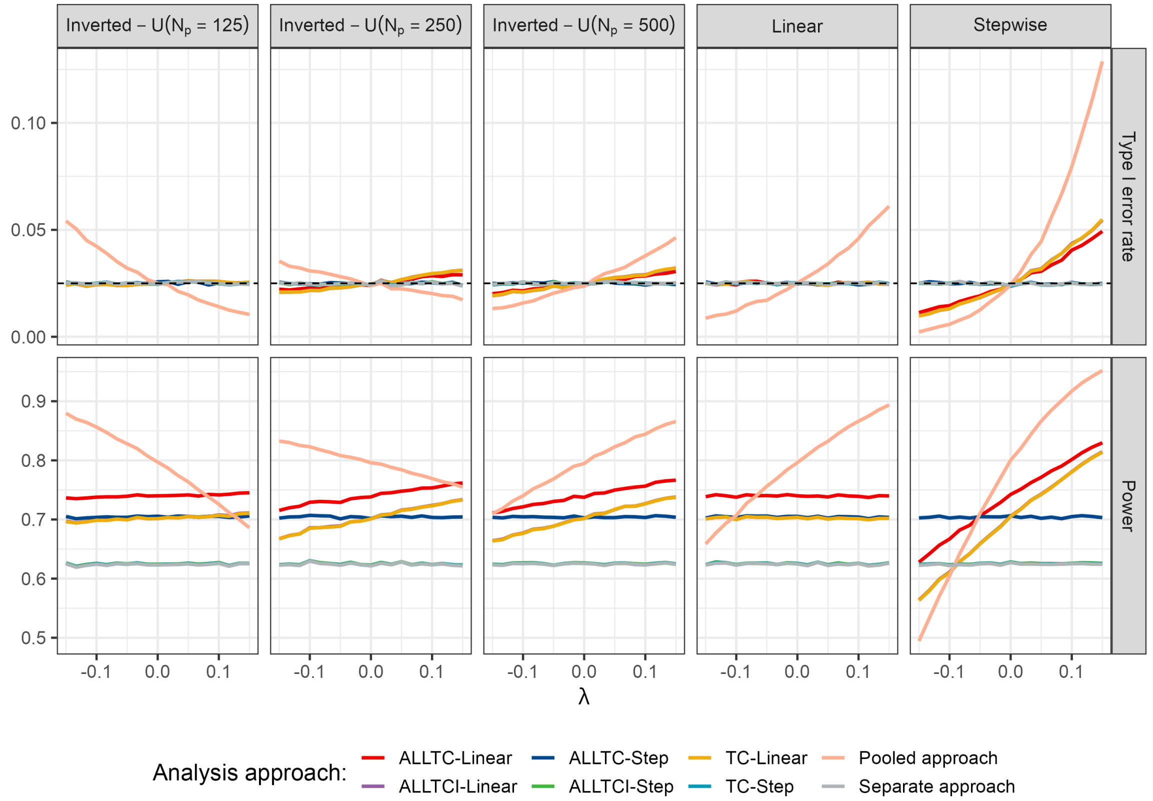

Figure S1: Type I error rate and power of rejecting H_{02} for continuous endpoints in the presence of equal linear, step-wise and inverted-U time trends across arms with respect to the strength of the time trend ($\lambda = \lambda_k$, $k = 0, 1, 2$) and according to the model used. Note that some lines overlap, e.g., ALLTC-Step and the separate approach are overlapping in the figures of the first row; ALLTCI-Step, TC-Step and the separate approach are overlapping in the figures of the second row.

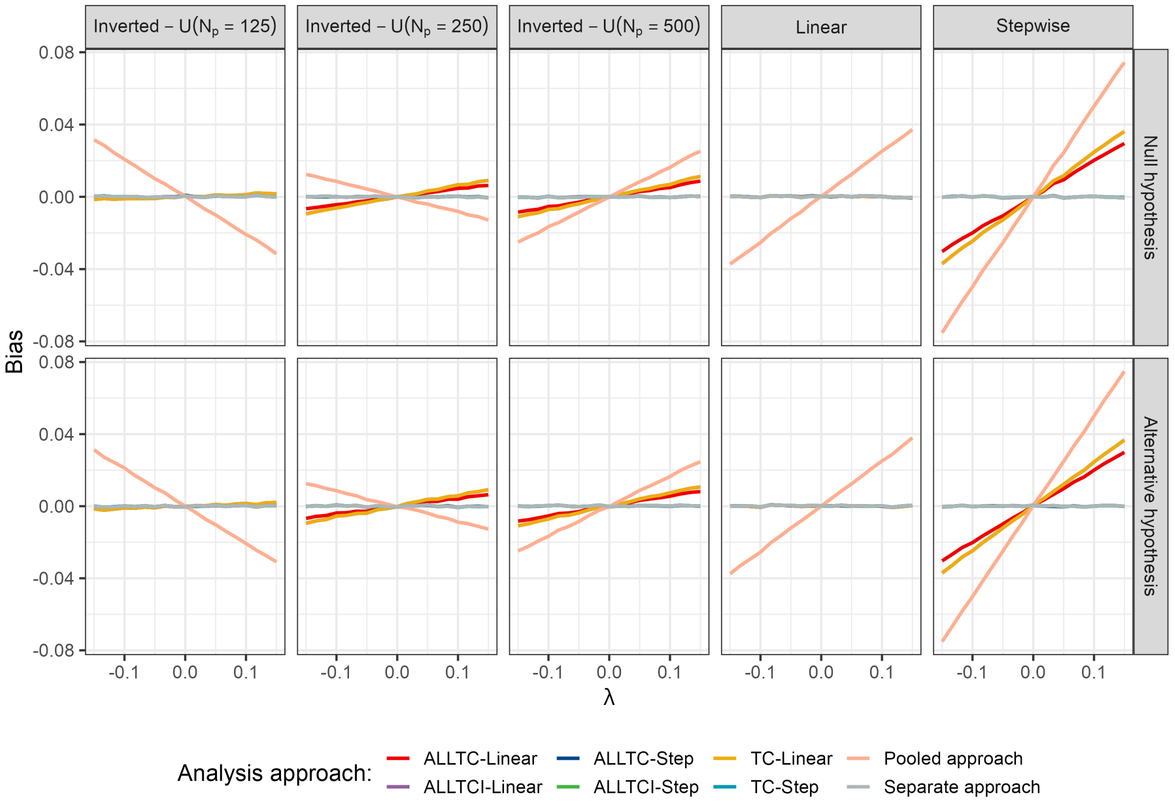

Figure S2: Bias of the treatment effect estimators (difference in means between control arm and treatment 2) for continuous endpoints in the presence of equal linear, step-wise and inverted-U time trends across arms with respect to the strength of the time trend ($\lambda = \lambda_k$, $k = 0, 1, 2$) and according to the model used.

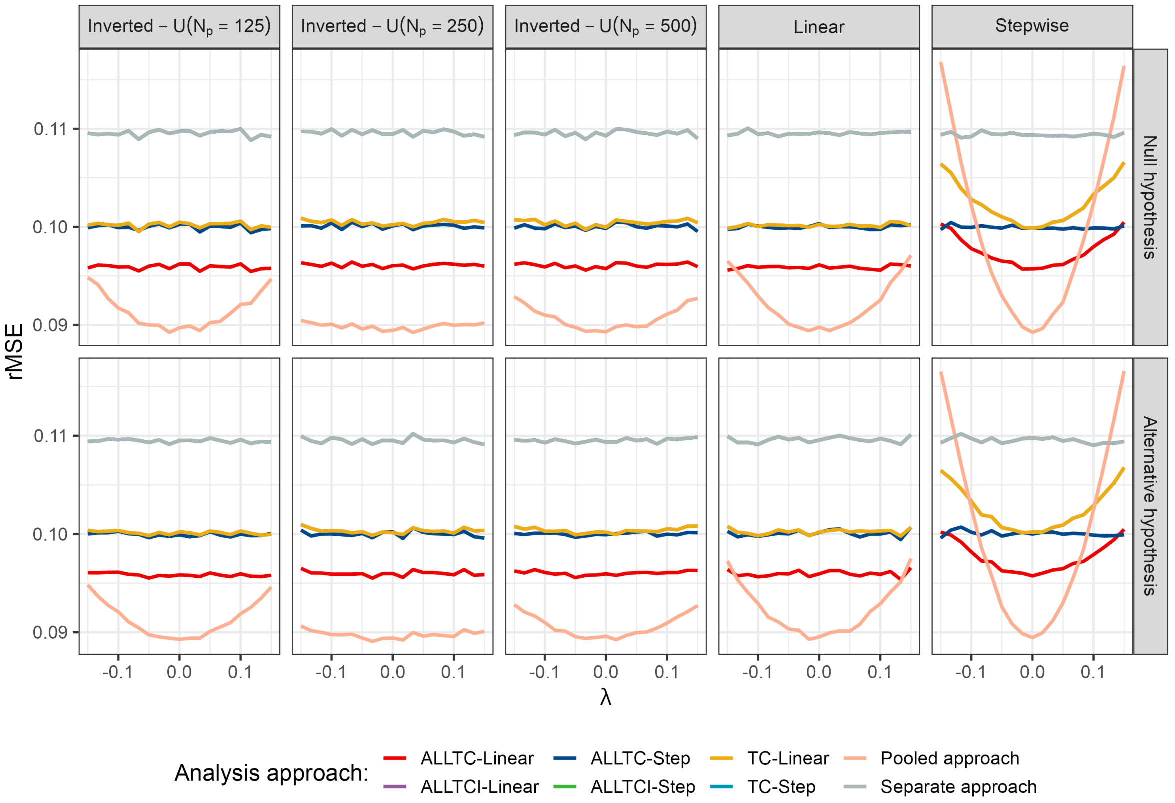

Figure S3: Root mean squared error of the treatment effect estimators (difference in means between control arm and treatment 2) for continuous endpoints in the presence of equal linear, step-wise and inverted-U time trends across arms with respect to the strength of the time trend ($\lambda = \lambda_k$, $k = 0, 1, 2$) and according to the model used.

E.1.2 Different time trends

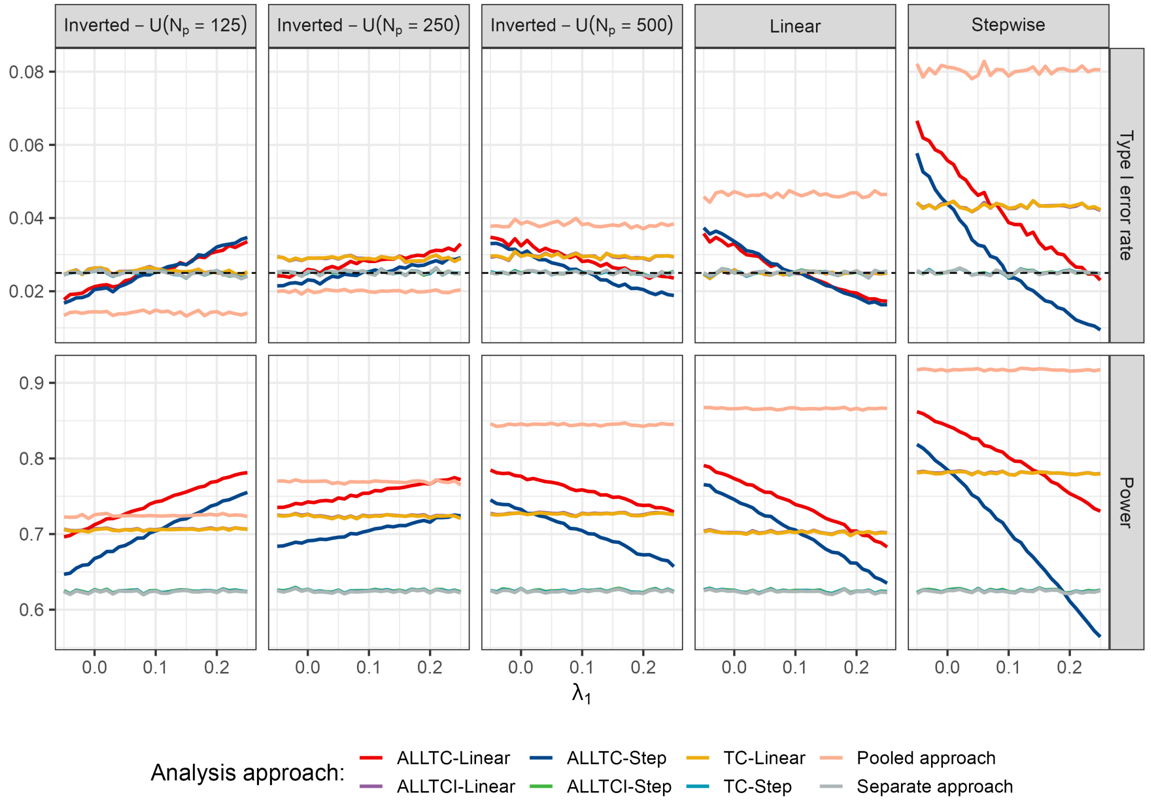

Figure S4: Type I error rate and power of rejecting H_{02} for continuous endpoints in the presence of different linear, step-wise and inverted-U time trends (for $\lambda_0 = \lambda_2 = 0.1$) with respect to the strength of the time trend in treatment arm 1 (λ_1) and according to the model used. Note that some lines overlap, e.g., ALLTCI-Step, TC-Step and the separate approach are overlapping in the figures of the second row.

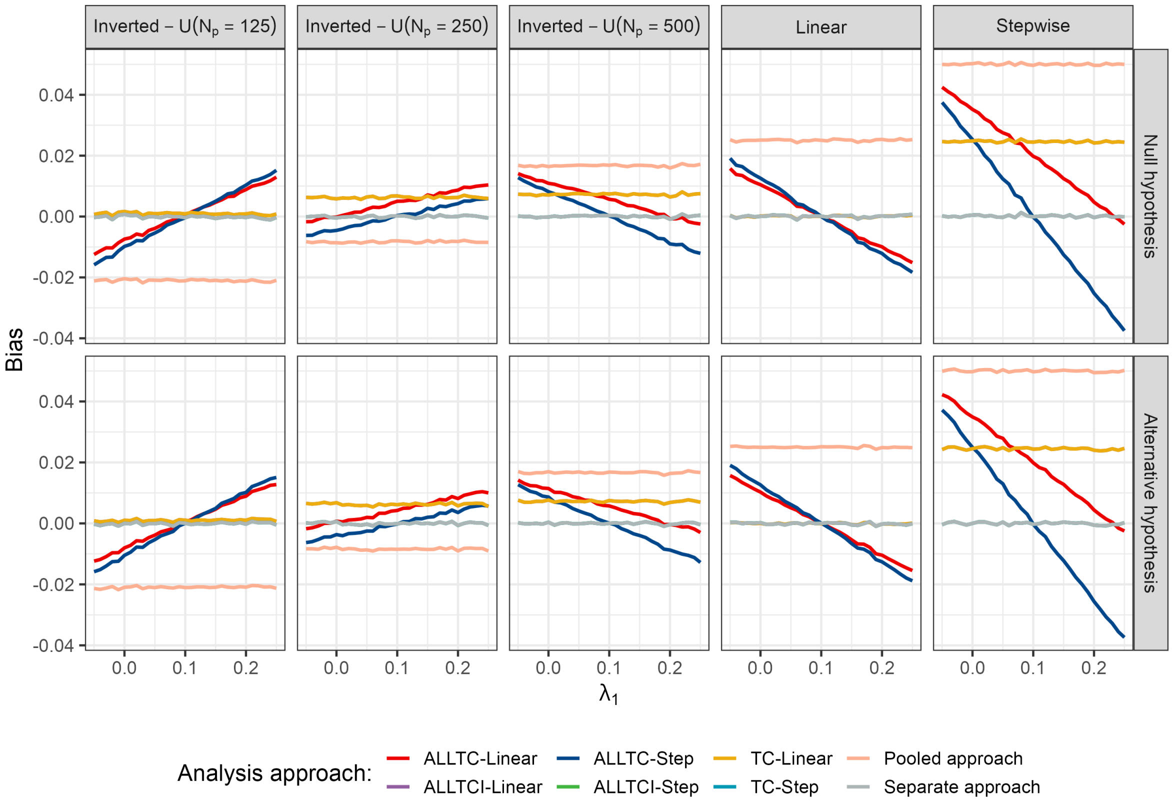

Figure S5: Bias of the treatment effect estimators (difference in means between control arm and treatment 2) for continuous endpoints in the presence of different linear, step-wise and inverted-U time trends (for $\lambda_0 = \lambda_2 = 0.1$) with respect to the strength of the time trend in treatment arm 1 (λ_1) and according to the model used.

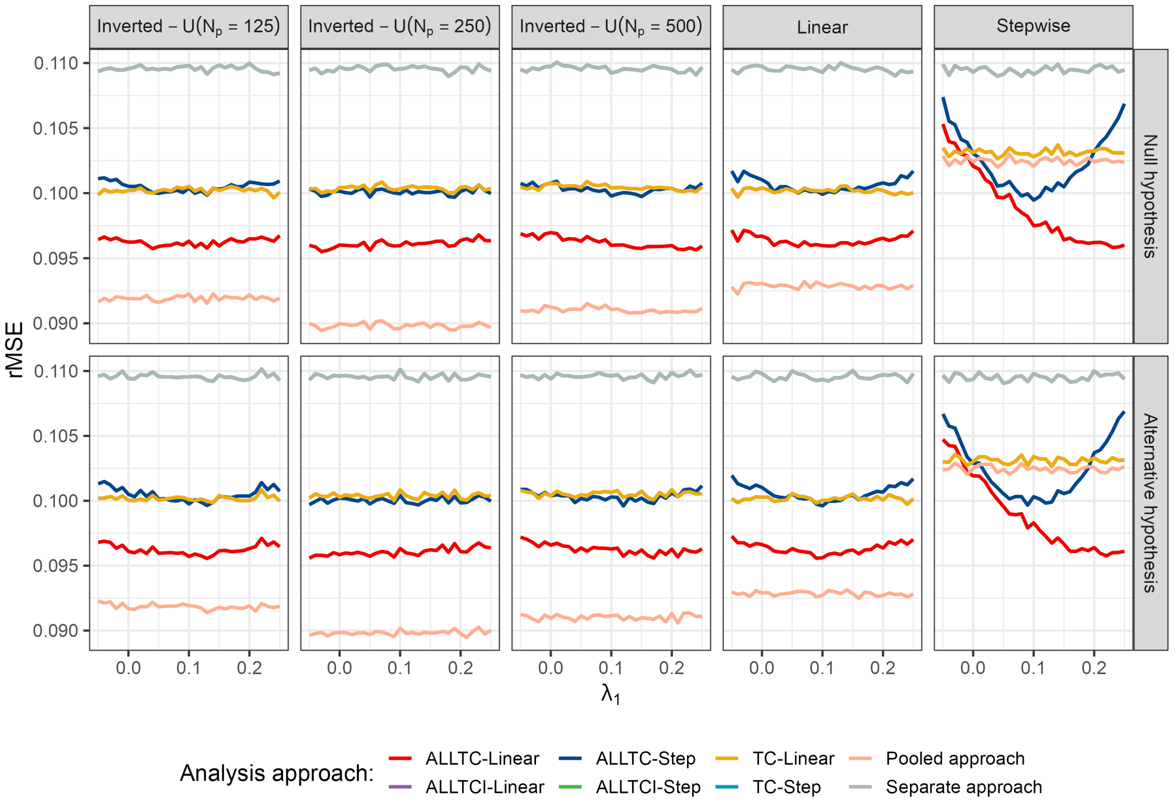

Figure S6: Root mean squared error of the treatment effect estimators (difference in means between control arm and treatment 2) for continuous endpoints in the presence of different linear, step-wise and inverted-U time trends (for $\lambda_0 = \lambda_2 = 0.1$) with respect to the strength of the time trend in treatment arm 1 (λ_1) and according to the model used.

E.2 Binary endpoints

Next, we present additional results for binary endpoints in the presence of time trends. As before, time trends can be either equal across all arms (Section E.2.1), or equal in the control group and treatment 2 but different in treatment 1 (Section E.2.2). We consider linear, step-wise and inverted-U time trend's patterns. As before, for the inverted-U trend, the trend switches in the middle of period 1 ($N_p = 125$), between the two periods ($N_p = 250$) or in the middle of period 2 ($N_p = 500$).

For scenarios with different time trends, we considered both, positive and negative trends for the control arm and treatment 2. Here, $\lambda_0 = \lambda_2$ were chosen in order to achieve 5% drop ($\lambda_0 = \lambda_2 < 0$) or increase ($\lambda_0 = \lambda_2 > 0$) in the response rate in the control arm from period 1 to period 2. Results for scenarios with positive time trend in the control and treatment 2 are shown in Figures S10, S12, S14 and S15. For scenarios with negative time trends in the control and treatment 2, the results are presented in Figures S11 and S13.

E.2.1 Equal time trends

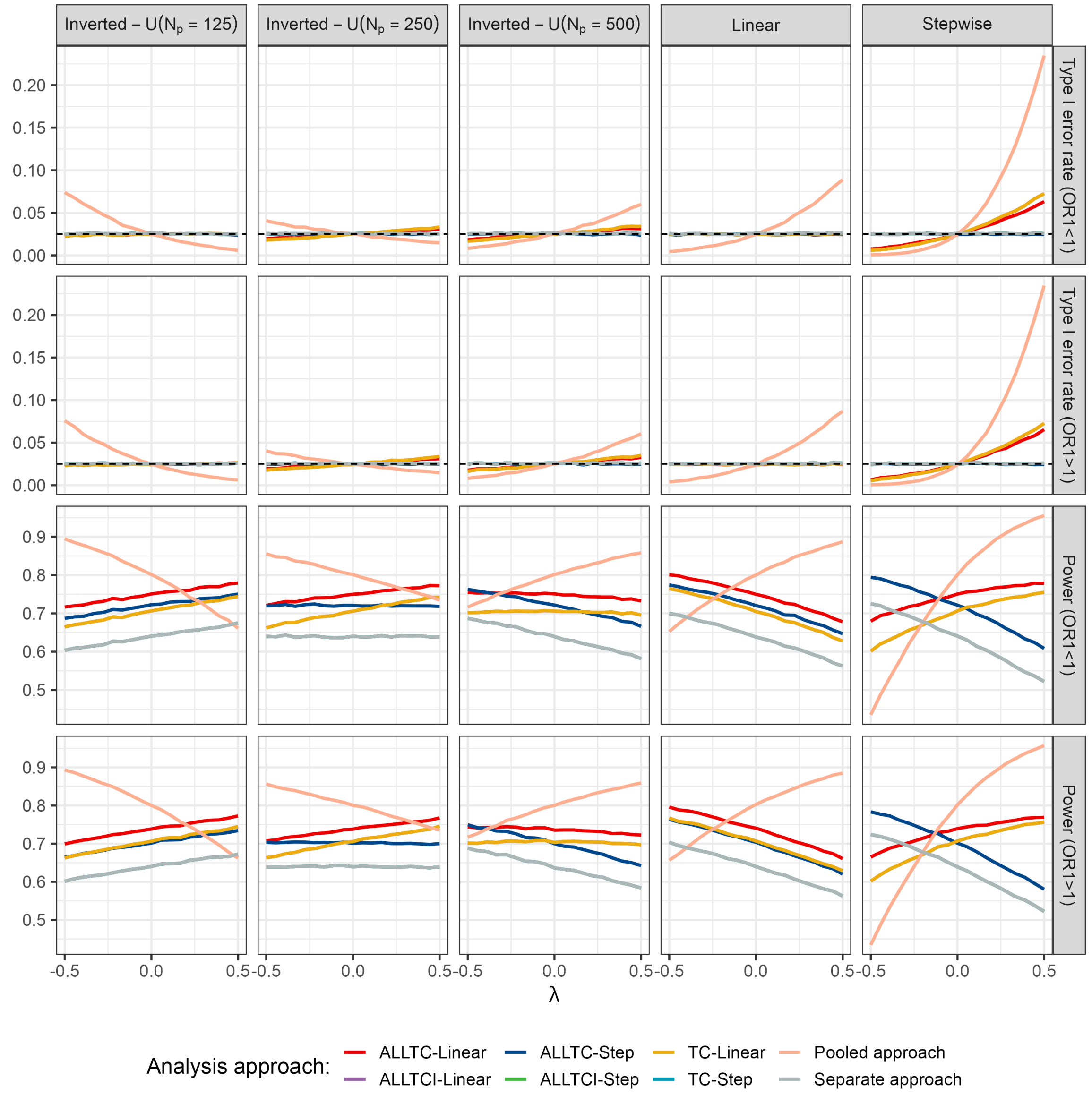

Figure S7: Type I error rate and power of rejecting H_{02} for binary endpoints in the presence of equal linear, step-wise and inverted-U time trends across arms with respect to the strength of the time trend ($\lambda = \lambda_k$, $k = 0, 1, 2$) and according to the model used. Note that some lines overlap, e.g., ALLTC-Step and the separate approach are overlapping in the figures of the first two rows.

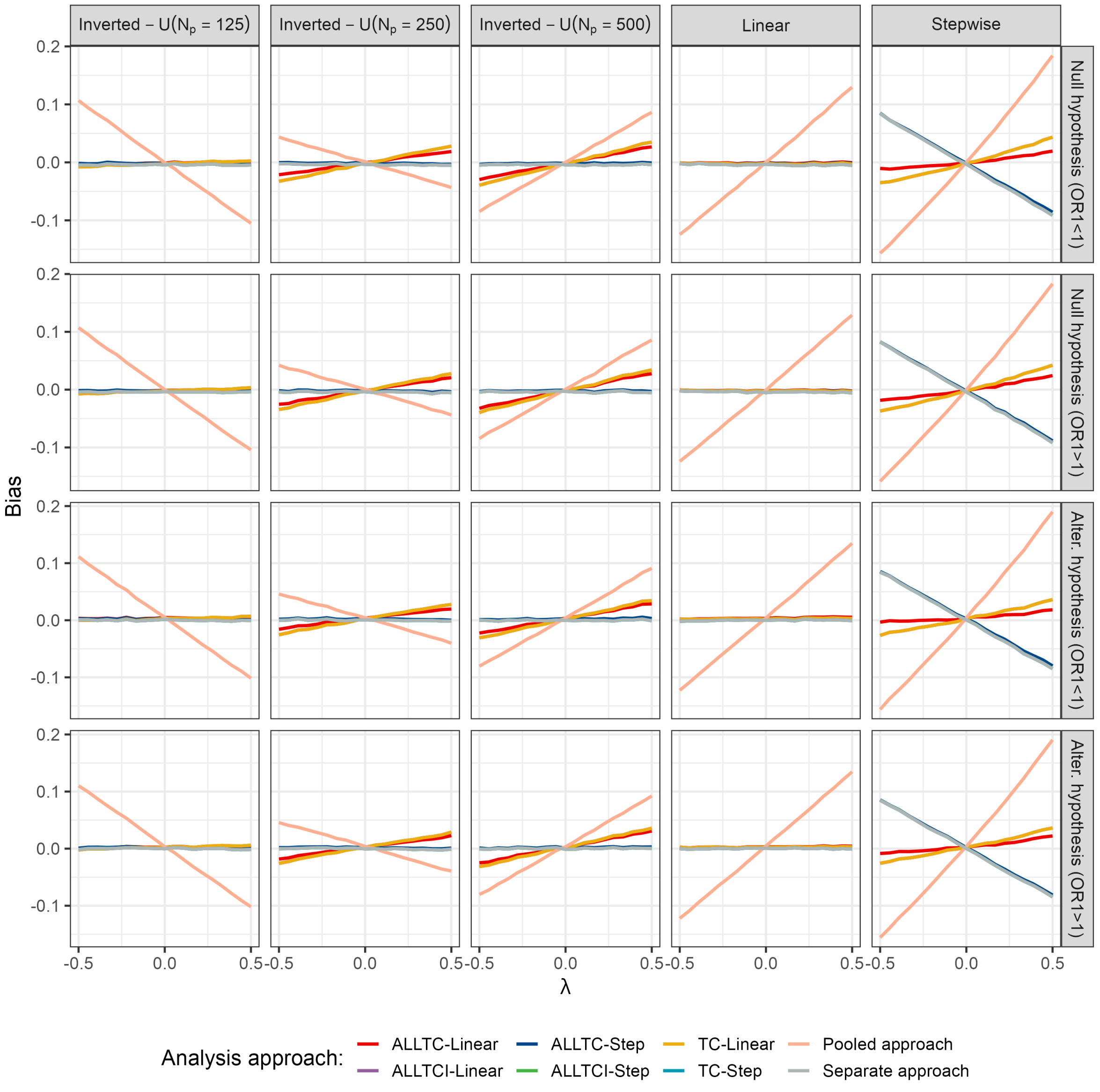

Figure S8: Bias of the treatment effect estimators ($\log(OR_2)$) for binary endpoints in the presence of equal linear, step-wise and inverted-U time trends across arms with respect to the strength of the time trend ($\lambda = \lambda_k$, $k = 0, 1, 2$) and according to the model used.

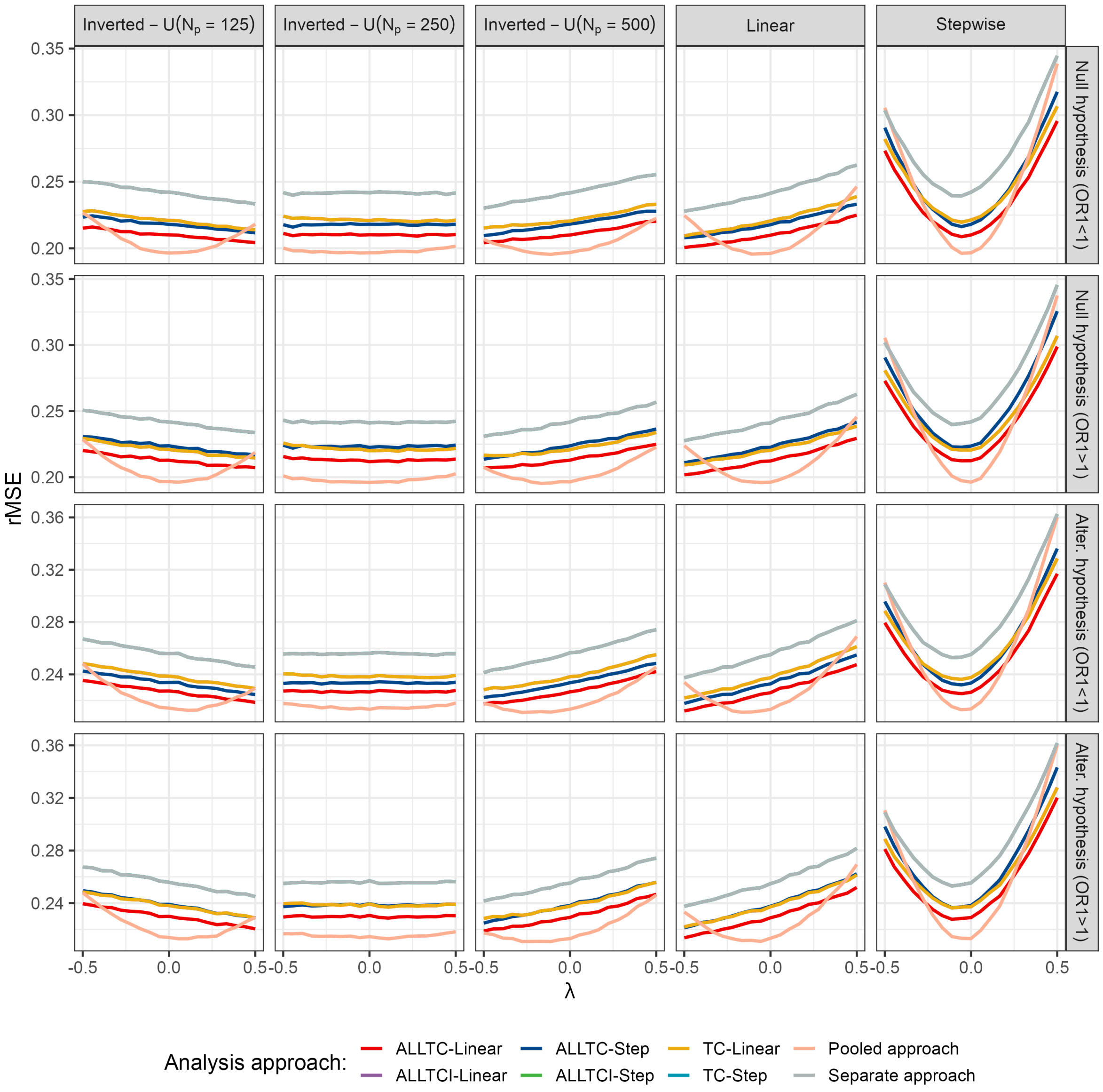

Figure S9: Root mean squared error of the treatment effect estimators ($\log(OR_2)$) for binary endpoints in the presence of equal linear, step-wise and inverted-U time trends across arms with respect to the strength of the time trend ($\lambda = \lambda_k$, $k = 0, 1, 2$) and according to the model used.

E.2.2 Different time trends

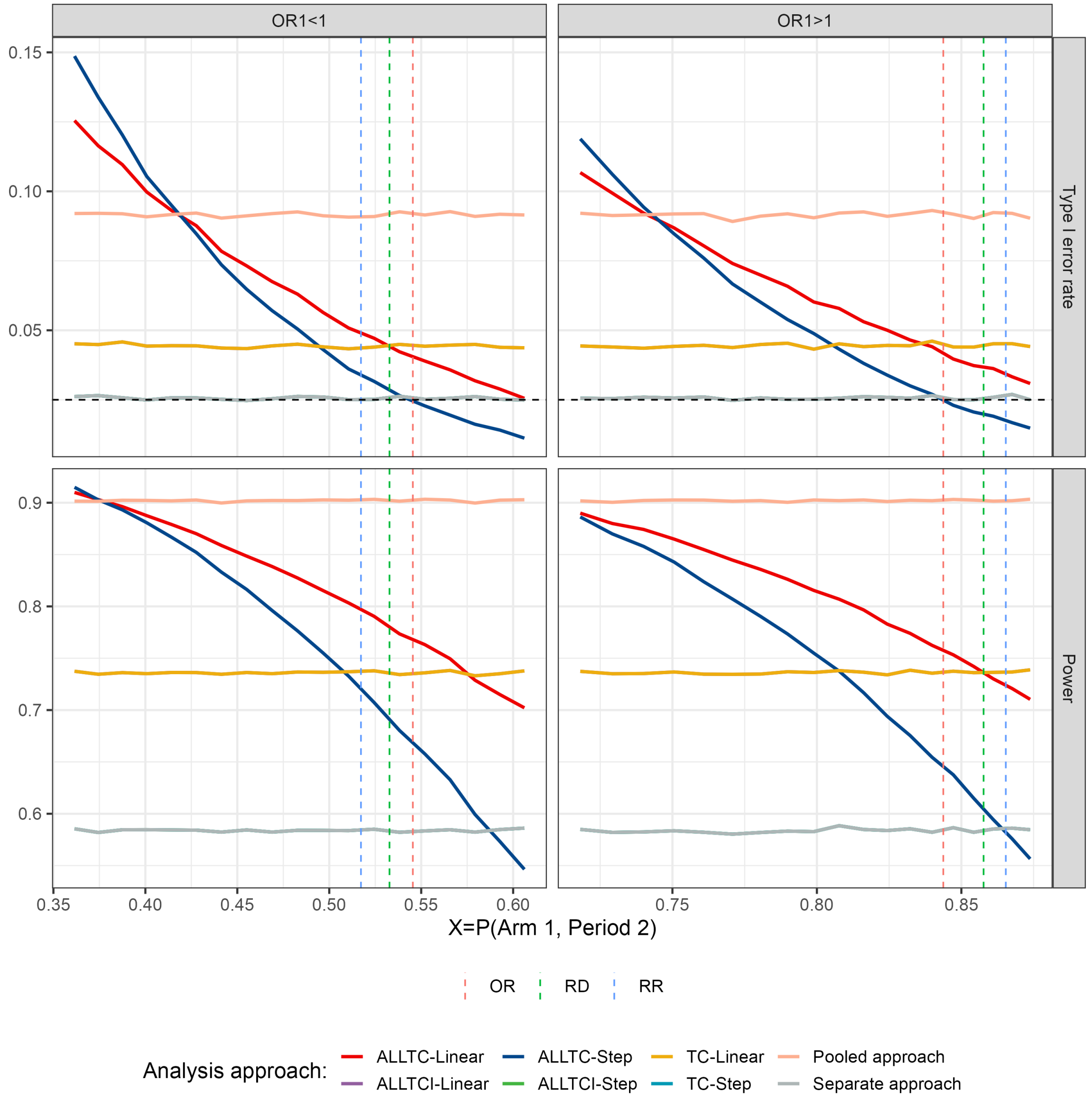

Figure S10: Type I error rate and power of rejecting H_{02} for binary endpoints in the presence of different step-wise time trends when there is a positive time trend in the control ($\lambda_0 = \lambda_2 > 0$ and varying λ_1) with respect to the response rate in treatment arm 1 in the second period, and depending on the model used. Note that some lines overlap, e.g., ALLTCI-Step, TC-Step and the separate approach are overlapping in the figures of the first and second rows.

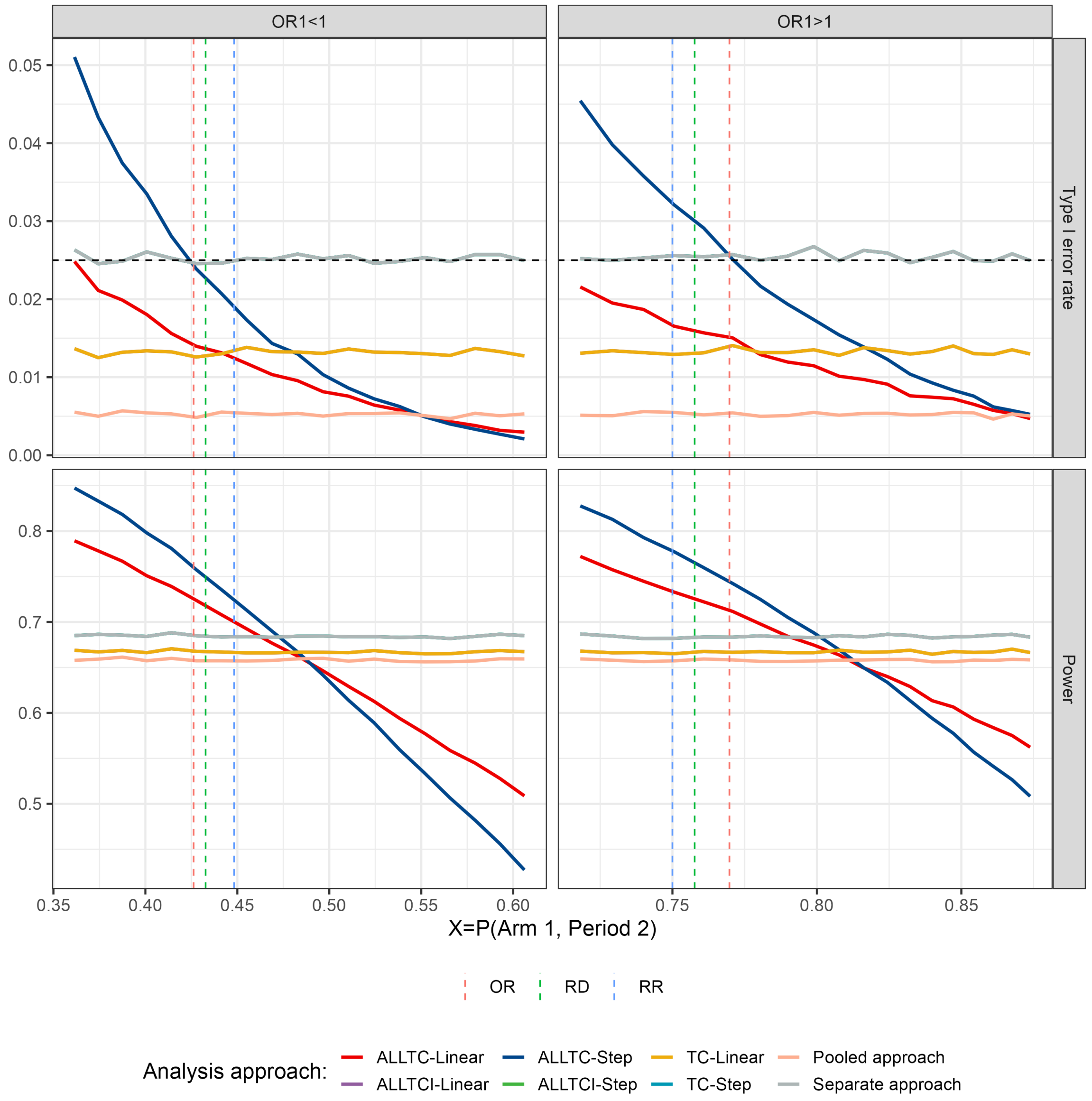

Figure S11: Type I error rate and power of rejecting H_{02} for binary endpoints in the presence of step-wise time trends when there is a negative time trend in the control ($\lambda_0 = \lambda_2 < 0$ and varying λ_1) with respect to the response rate in treatment arm 1 in the second period, and depending on the model used. Note that some lines overlap, e.g., ALLTCI-Step, TC-Step and the separate approach are overlapping in the figures of the first and second rows.

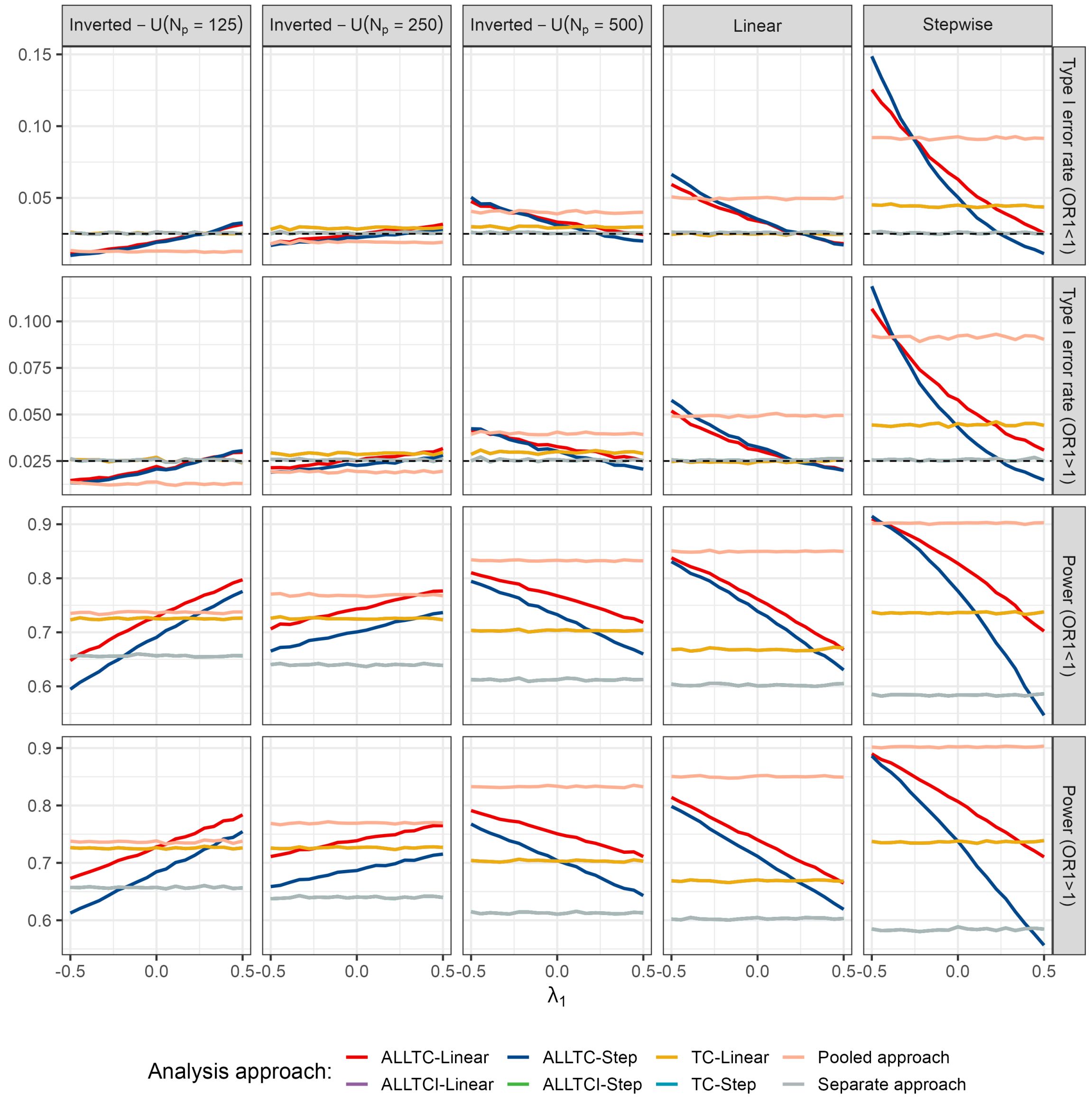

Figure S12: Type I error rate and power of rejecting H_{02} for binary endpoints in the presence of different linear, step-wise and inverted-U time trends when there is a positive time trend in the control ($\lambda_0 = \lambda_2 > 0$) with respect to the strength of the time trend in treatment arm 1 (λ_1) and according to the model used. Note that some lines overlap, e.g., ALLTCI-Step, TC-Step and the separate approach are overlapping in the figures of the first and second rows.

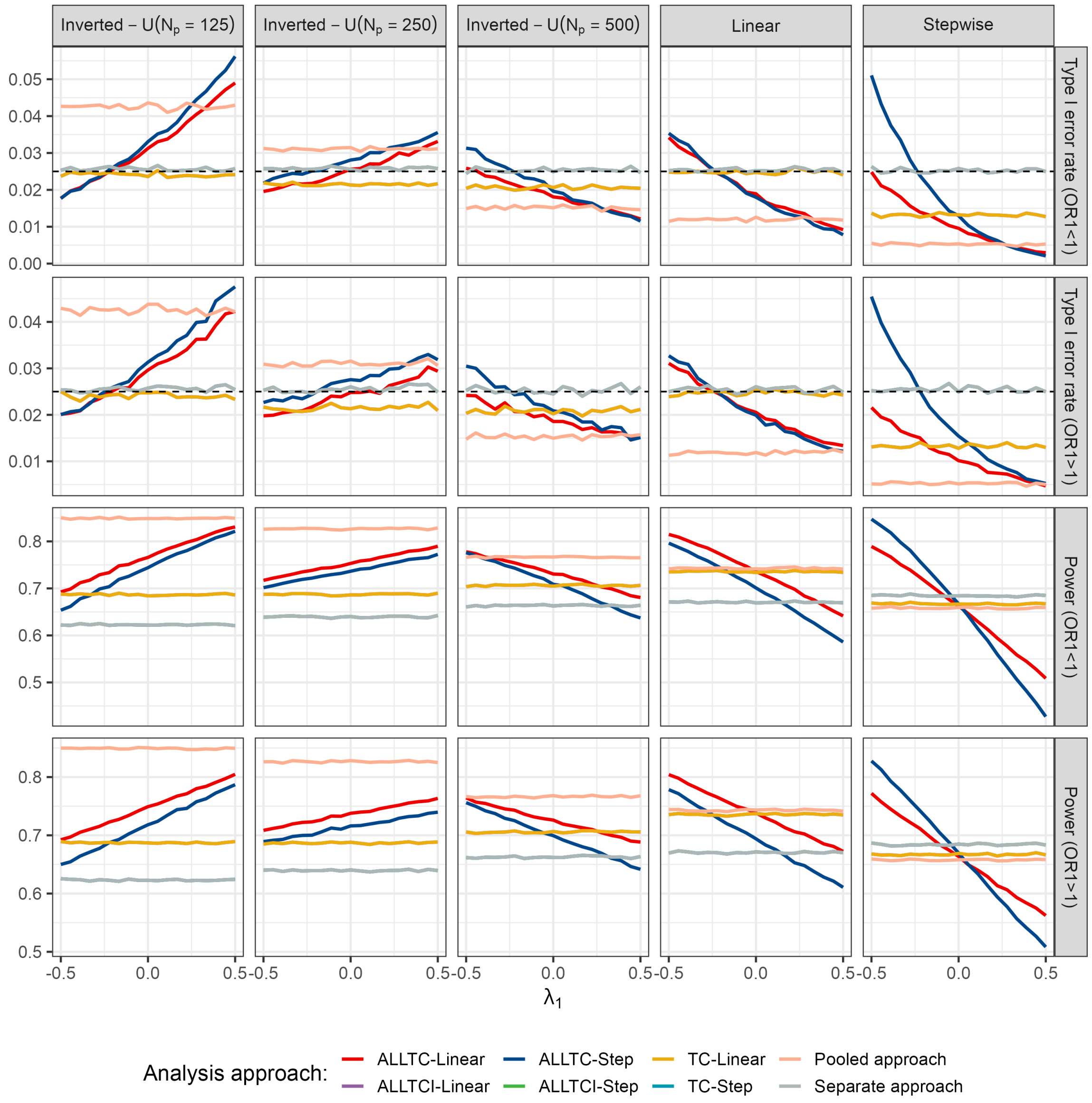

Figure S13: Type I error rate and power of rejecting H_{02} for binary endpoints in the presence of linear, step-wise and inverted-U time trends when there is a negative time trend in the control ($\lambda_0 = \lambda_2 < 0$) with respect to the strength of the time trend in treatment arm 1 (λ_1) and according to the model used. Note that some lines overlap, e.g., ALLTCI-Step, TC-Step and the separate approach are overlapping in the figures of the first and second rows.

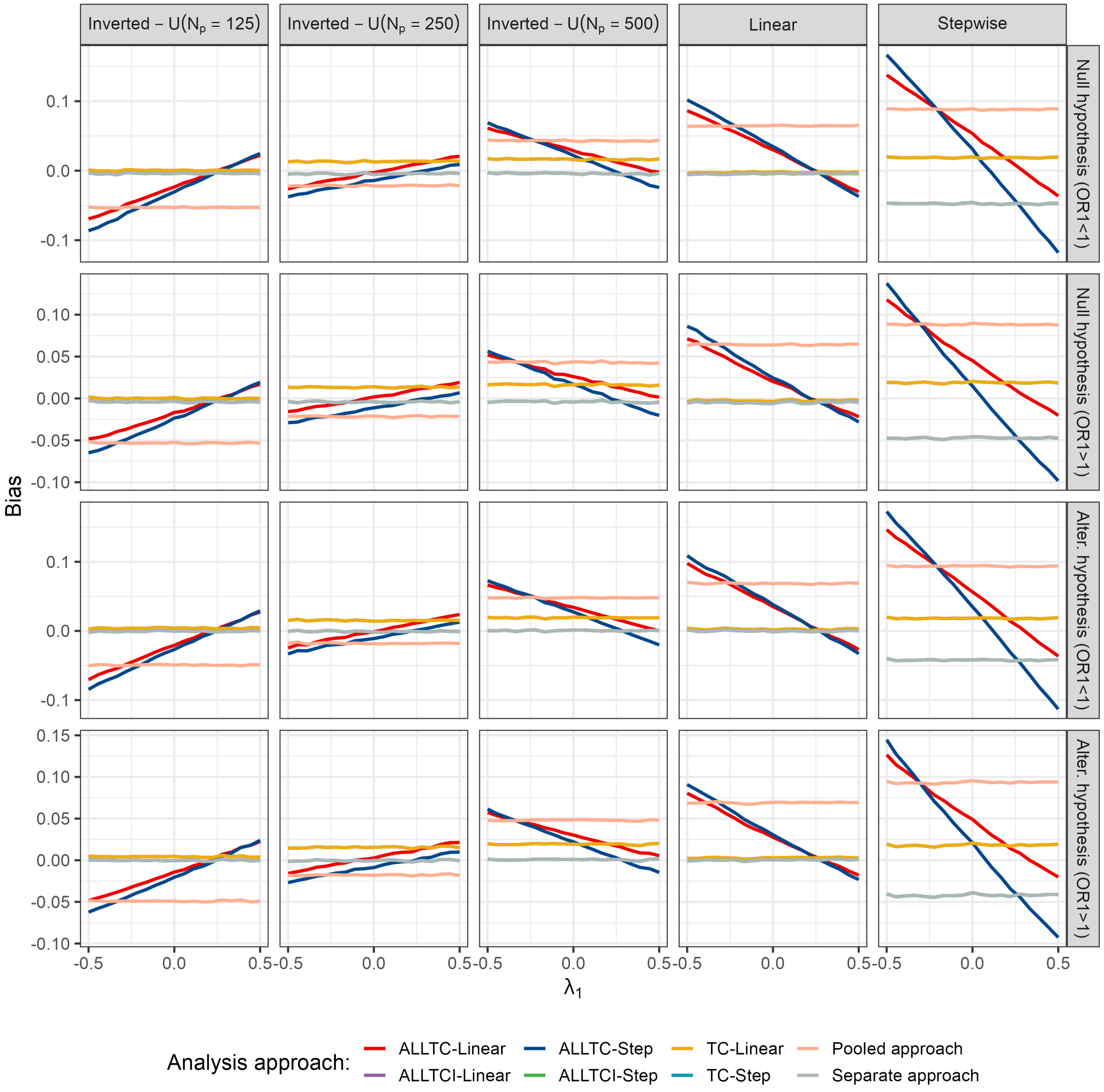

Figure S14: Bias of the treatment effect estimators ($\log(OR_2)$) for binary endpoints in the presence of linear, step-wise and inverted-U time trends when there is a positive time trend in the control ($\lambda_0 = \lambda_2 > 0$) with respect to the strength of the time trend in treatment arm 1 (λ_1) and according to the model used.

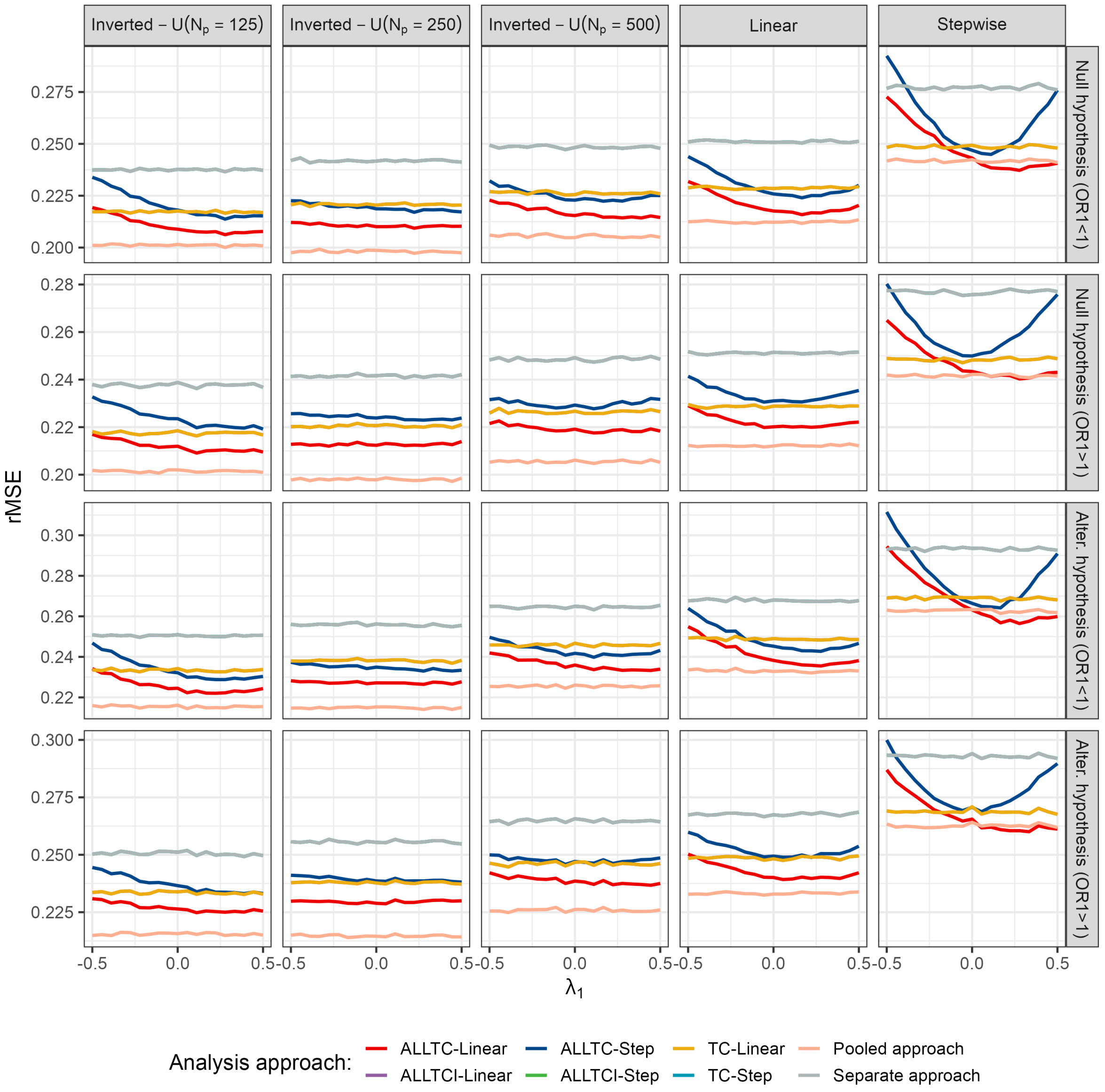

Figure S15: Root mean squared error of the treatment effect estimators ($\log(OR_2)$) for binary endpoints in the presence of linear, step-wise and inverted-U time trends when there is a positive time trend in the control ($\lambda_0 = \lambda_2 > 0$) with respect to the strength of the time trend in treatment arm 1 (λ_1) and according to the model used.

F Additional simulations

F.1 Comparison of randomization procedures and patient entry time schemes

In this section, we compare different randomization procedures through simulations and discuss their role when incorporating non-concurrent controls. In particular, we consider two randomization procedures, simple randomization and block randomization [1], and study their impact on the type I error rate. In addition, we evaluate the impact of patient entry times when these are random rather than deterministic as considered in the article.

F.1.1 Data generation

We simulated data of a two-period platform trial as described in Section 2 of the paper. In this case, however, we assumed that time trends are equal across groups and additive on the model scale such that the data are generated according to the model:

$$g\left(E(Y_j)\right) = \eta_0 + \sum_{k=1,2} \theta_k \cdot I(k_j = k) + f(t_j), \tag{6}$$

where Y_j, $g()$, η_0 and θ_{k_j} refer to the continuous or binary response, the link function (identity and logit functions for continuous or binary responses, respectively), the control response and treatment effects, respectively.
In this section, we considered a scenario in which there is no trend in the first period and the trend starts in the second period and is linear. Specifically, the time trend $f(t_j)$ is assumed to have the following pattern:

$$f(t_j) = \begin{cases} \lambda \frac{t_j - 1}{N-1} & j > N_1 \\ 0 & j \leq N_1, \end{cases} \tag{7}$$

where N_1 denotes the sample size in the first period and N is the total sample size in the trial, so that the examined pattern corresponds to no time trend in the first period and linear time trend of strength λ for all arms in the second period. Cases with moderate ($\lambda = 0.15$) and extreme ($\lambda = 5$) strengths of the time trend are examined.
For the patient entry times t_j, two options are discussed:

- deterministic entry times: $t_j = j$
- random entry times: $t_j \sim U(0, N_1/N)$ in the first period and $t_j \sim U(N_1/N, 1)$ in the second period

For deterministic entry times, the order of patients' entry in the trial is the same as that of the randomization sequence. In every unit of time, a patient enters into the platform. Thus, patients' index is equivalent to time at which patients enter in the study. Random entry times are uniformly distributed in both periods. For comparative purposes, random times are multiplied by the total number of sample size N to achieve the same scale as in the case with deterministic entry times.
Furthermore, we assumed that the null hypothesis holds for treatment arm 2 (i.e. $\theta_2 = 0$ and $OR_2 = 1$). For continuous endpoints, we used control response $\eta_0 = 0$ and effect size for treatment 1 $\theta_1 = 0.25$. For binary endpoints, the control response rate was set to 0.3 and odds ratio for treatment 1 to 1.8. We considered a significance level $\alpha = 0.025$ to test the null hypothesis for treatment 2 against the one-sided alternative.
The ALLTC-Step model, i.e. model using all treatment data and control and adjusting for time by a step function, is used for the evaluation of the data. Each scenario was replicated 100.000 times.

F.1.2 Randomization procedures

Simple randomization per period. The randomization procedure corresponds to the random allocation rule in which the sample size per arm in each period is prespecified. Thus, in each period, we take a random sample of the possible treatment allocation combinations given the sample sizes per arm and per period. So that this sequence represents the treatment allocation for each patient.

Block randomization. We considered the randomization procedure to be block randomization. Patients are assigned to arms following blocked randomization per period. In the first period (in which we have allocation ratio 1:1), we consider a block size of 4, while in the second period (in which we have 1:1:2) a block size of 12.

F.1.3 Results

Simple randomization per period. For continuous endpoints, simple randomization maintains the type I error at 2.5% in the presence of moderate time trends for both, deterministic and random entry times. However, if the time trends have extreme strength, the type I error rate is inflated, reaching an empirical level of 3.3% for deterministic and random entry times.

In the setting with binary endpoints, the type I error rate is maintained, despite the strength of the time trend. That is, even when the time trend is extreme and regardless of whether the times were random or deterministic.

Block randomization. Similarly as in previous scenarios, for continuous endpoints when using block randomization, the type I error rate is maintained at 2.5% for fixed and random entry times whenever the time trend is moderate. In the presence of extreme time trends, however, this randomization procedure leads to strictly conservative results and empirical type I error rate of 0.7% for both, fixed and random entry times. For binary endpoints, the type I error rate is again maintained at 2.5% for moderate and extreme time trend strengths and deterministic and random entry times.

F.2 Platform trials with three periods

In this section, we investigate the performance of the proposed models (1) and (2) in a trial with two treatment arms and a shared control in which arm 2 enters when the trial is ongoing. However, unlike the trial design reported in the article, here arm 1 finishes before arm 2 does. Hence, in this section, the platform trial is divided into three periods (instead of two periods as in the paper), where treatment arm 1 is active in the first 2 periods and treatment arm 2 in periods 2 and 3 (see Figure S16 for an illustration).

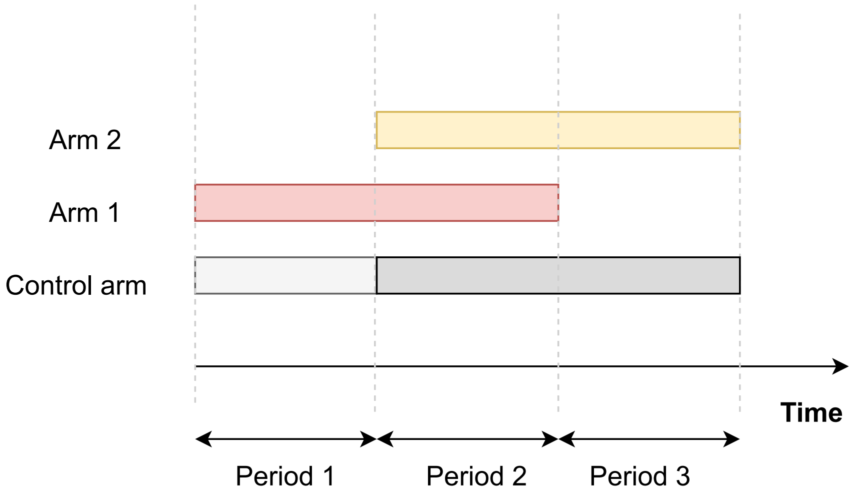

Figure S16: Scheme of a platform trial with 3 periods. Non-concurrent controls for arm 2 are shown in light grey.

F.2.1 Data generation

We simulated data using the generating model (4) in the paper and following the same procedure than the one described in section F.1.1. We assumed linear time trend pattern, with the trend function $f(\cdot)$ given by:

$$f(j) = \lambda_{k_j} \frac{(j-1)}{(N-1)}$$

We investigated cases with equal strength of the time trend across all arms (i.e., $\lambda = \lambda_0 = \lambda_1 = \lambda_2$) as well as cases where the time trend in arm 1 differs (i.e., $\lambda_0 = \lambda_2 \neq \lambda_1$).
As in the main paper, we assumed that treatment 2 joins the platform after 1/2 of the patients have been allocated to treatment arm 1 and considered equal sample sizes n in both treatment arms. In the newly considered scenarios, however, we used allocation ratio 1:1:1 in each period, thus treatment 1 leaves the trial earlier. Note that this results in larger sample size in the control group, as it is present in all 3 periods. Patients were assigned to arms according to block randomisation with block sizes of $2 \cdot (\#\text{active arms} + 1)$ in each period.

For continuous endpoints we assumed control response $\eta_0 = 0$, treatment effect for arm 1 of $\theta_1 = 0.25$ and treatment effects for arm 2 of $\theta_2 = 0$ or $\theta_2 = 0.25$ under the null and alternative hypotheses, respectively. In scenarios with binary endpoints, control response rate of $\eta_0 = 0.7$ and odds ratio for treatment 1 of 1.8 were used in all cases. Odds ratio for treatment 2 was set to 1 under the null hypothesis and to 1.8 under the alternative. We considered the sample size per treatment arm of $n = 210$, as this results in 80% power for the pooled analysis to detect the difference between the control group and treatment 2, assuming the given treatment effects, at 2.5% one-sided significance level in the case of no time trend.

F.2.2 Results

The results of these additional simulations coincide with those presented in the main paper. In particular, under equal time trends, the power achieved using model (1) is higher than the power using model (2) and the separate approach, while the type I error is still controlled. If the time trend in treatment arm 1 differs from that in treatment arm 2 and control, the type I error rate is no longer maintained at 0.025. The type I error rate inflation, however, is less pronounced in the newly considered scenarios with three periods. This results from the fact that as there is now less overlap between the two treatments, treatment arm 1 contributes less to the estimation of the time trend and this estimation is then less affected by the different time trend in this arm.

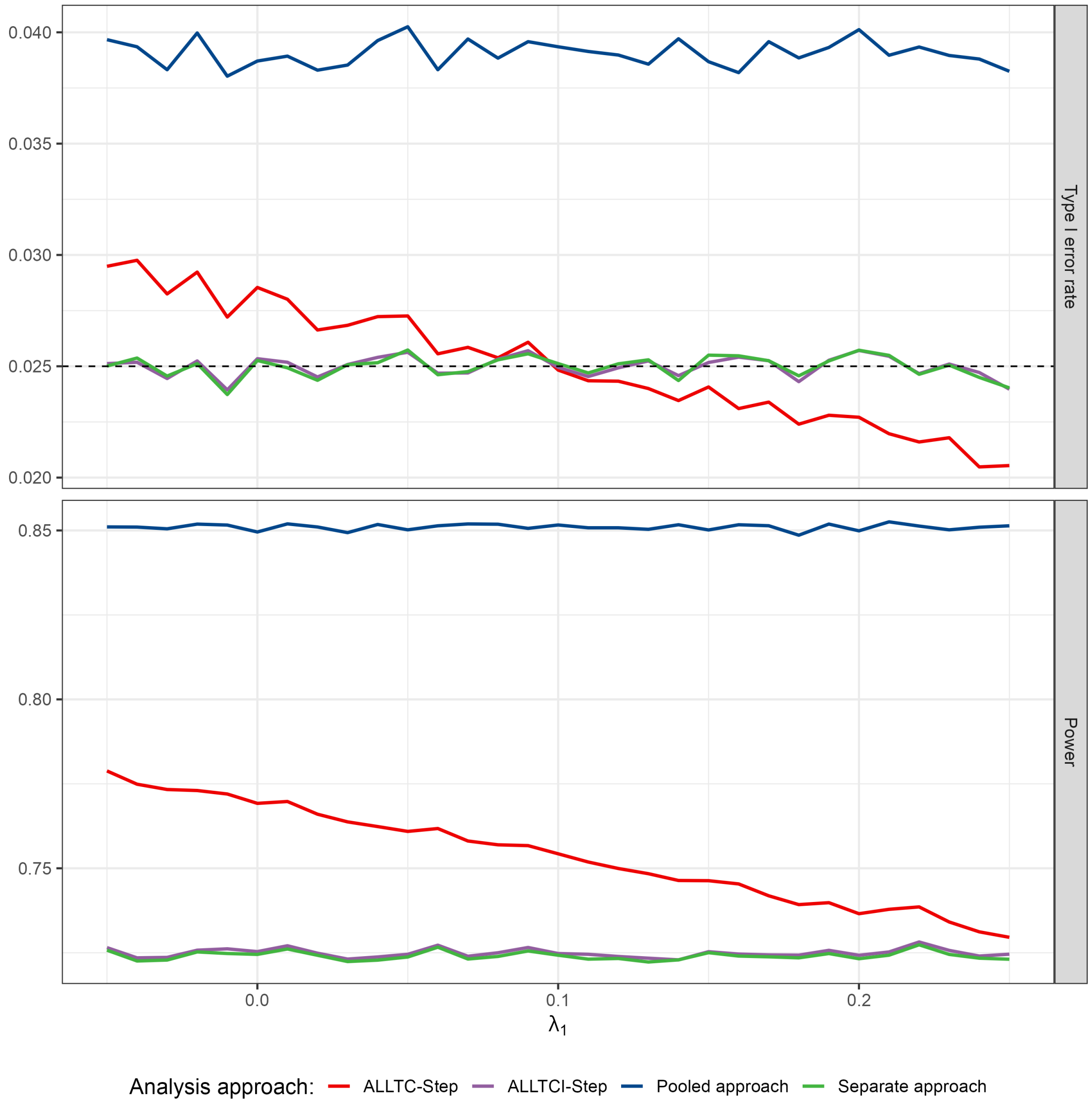

Figure S17: Type I error rate and power of rejecting H_{02} for continuous endpoints in the presence of different linear time trends (for $\lambda_0 = \lambda_2 = 0.1$) with respect to the strength of the time trend in treatment arm 1 (λ_1) and according to the model used.

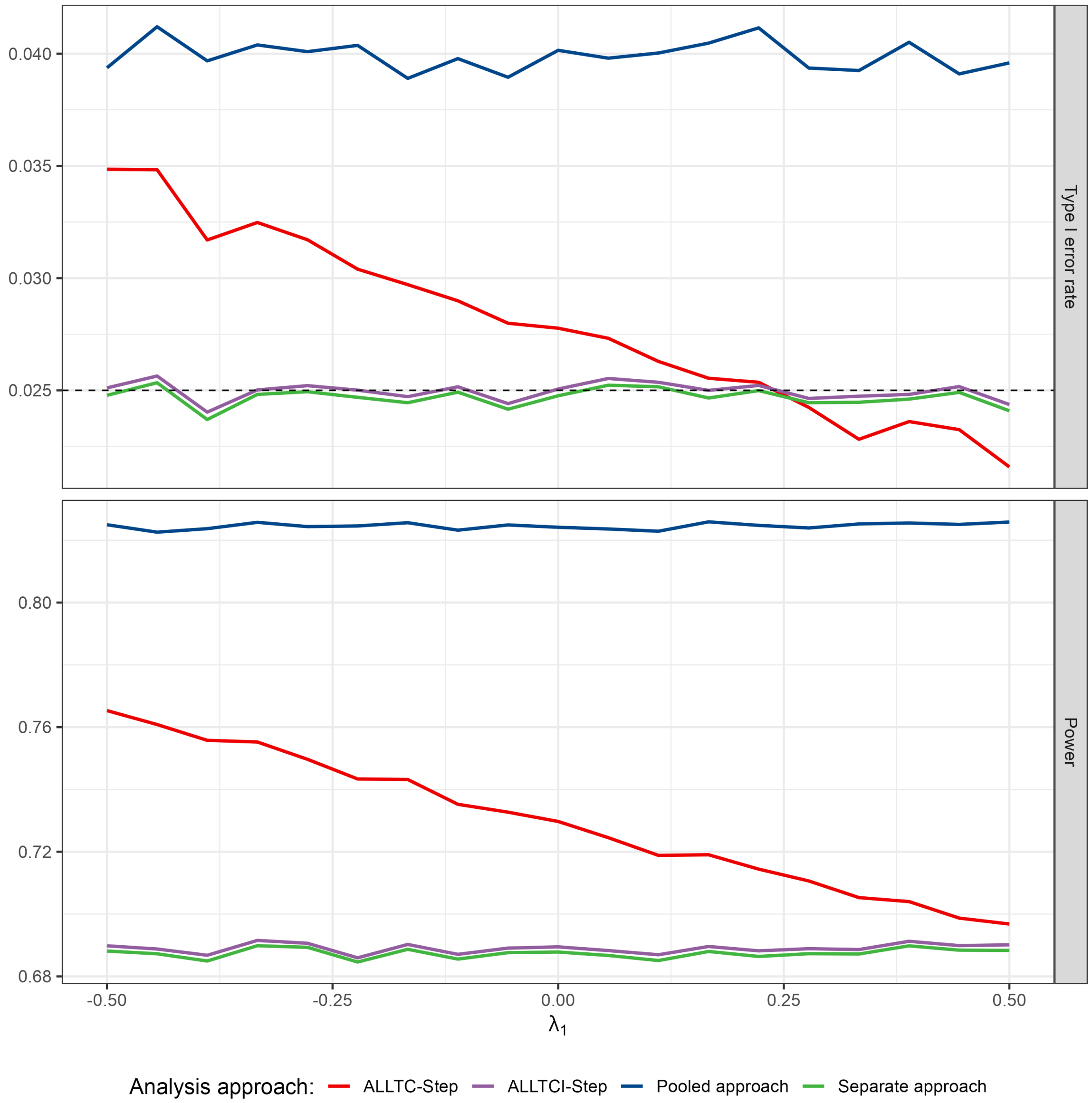

Figure S18: Type I error rate and power of rejecting H_{02} for binary endpoints in the presence of different linear time trends when there is a positive time trend in the control ($\lambda_0 = \lambda_2 > 0$) with respect to the strength of the time trend in treatment arm 1 (λ_1) and according to the model used.

References

[1] Lachin, J. M. (1988). Statistical properties of randomization in clinical trials. Controlled Clinical Trials, 9(4), 289-311.
